# Impact of Emotional Labor and Positive Psychological Capital on the Turnover Intention of Nurses Caring for Patients with COVID-19: A Descriptive Survey Study

**DOI:** 10.1155/2024/5517249

**Published:** 2024-01-08

**Authors:** Mira Kwon, Yeoungsuk Song

**Affiliations:** ^1^Kyungpook National University Hospital, Clinical Trial Center, Daegu, Republic of Korea; ^2^Kyungpook National University College of Nursing, Research Institute of Nursing Science, Daegu, Republic of Korea

## Abstract

Nurses' turnover intention has increased since the COVID-19 pandemic. Emotional labor is reportedly high among nurses in Korea, and a positive psychological capital can help reduce turnover intention. This cross-sectional study investigated the factors influencing turnover intention in nurses during the COVID-19 pandemic. Survey data were collected from 155 nurses caring for patients with COVID-19 at a university hospital in South Korea in March 2022. Self-reported and paper-based questionnaires on emotional labor, positive psychological capital, and turnover intention were employed. The mean values for emotional labor, positive psychological capital, and turnover intention were 54.52/80, 58.03/90, and 38.92/50, respectively, and 77.8% of nurses reported planning to quit working. Turnover intention correlated with emotional labor (*r* = 0.17; *p*=0.041) and had no significant relationship with positive psychological capital. Sex (*β* = 0.24; *p*=0.003) and emotional labor (*β* = 0.18; *p*=0.019) affected turnover intention, with 9% explanatory power. Female nurses caring for patients with COVID-19 had a higher turnover intention than male nurses. Finally, the higher the emotional labor of nurses caring for patients with COVID-19, the higher the turnover intention. To reduce the turnover intention of nurses, hospitals need to help them enhance their emotional labor and positive psychological capital by establishing measures such as emotional coaching programs or psychological capital interventions.

## 1. Introduction

Coronavirus disease (COVID-19) is an infectious respiratory disease caused by the SARS-CoV-2 virus [1]. The current COVID-19 pandemic has placed greater burdens on the already exhausted nursing workforce, acting as a strong determinant of nurse resignation [2]. Accordingly, nurses' turnover intention has drastically increased since the pandemic outbreak [3], with a higher level in those caring for patients with COVID-19 than in those in general wards [4]. The International Council of Nurses (ICN) also noted the worsening high turnover rate of nurses and the shortage of nursing staff due to the COVID-19 pandemic, saying governments are working on mitigating the risk of increased turnover among nurses and improving nurse retention [5]. Along with these efforts, it is necessary to make efforts to lower nurses' turnover intention by identifying the factors affecting such intention among nurses caring for patients with COVID-19.

Nurses in the front line against the COVID-19 pandemic faced substantial changes in their workplace and experienced profoundly emotional labor in the care process of patients with COVID-19 [6]. Emotional labor refers to efforts to control one's actual emotions for effective job performance and to express specific emotions required by organizational norms [7]. Given that nurses constantly communicate with patients, they have to manage their own actual feelings [8]. The levels of nurses' emotional labor were above the average during this pandemic [8]. Higher levels of emotional labor were associated with higher levels of nurses' turnover intention, and emotional labor was the strongest impact factor on turnover intention [9]. Therefore, in order to identify the factors to lower the turnover intention of nurses in charge of COVID-19 patients, it is necessary to investigate the effect of emotional labor of nurses in charge of patients with COVID-19 on turnover intention.

Nurses in the front line of the fight against COVID-19 are at risk of contagion and death, as well as encounter with a complex and unexpected scenario, which experiences the exposure of the nurses to difficulties that may hinder their emotional wellbeing [6]. A positive psychological capital implies a positive mental state that allows a person to achieve goals and improve performance according to individual psychological strengths [10]. Positive psychological capital has four dimensions: self-efficacy, optimism, resilience, and hope [11]. It also contributes to improving organizational performance, job satisfaction, and life satisfaction [12] and is a primary variable that reduces anxiety and depression experienced by healthcare professionals during the pandemic [13]. In 4865 Chinese nurses, psychological capital was a negative predictor of turnover intention; the higher the psychological energy, the more stable and the lower their intention to leave [14]. In other words, increasing the positive psychological capital contributes to greater job retention. Based on this background, there is a possibility that positive psychological capital may affect the turnover intention of nurses in charge of COVID-19 patients in Korea. Therefore, investigating the impact factor of positive psychological capital of nurses caring for patients with COVID-19 on turnover intention in Korea may be important.

During the COVID-19 pandemic, several studies have been conducted on nurses' turnover intention [2–4, 15], emotional labor [6, 8, 9], and positive psychological capital [14, 16, 17] separately. The studies described various emotional states and emotional labor experienced by nurses during the COVID-19 pandemic [6, 8] and the relationship between emotional labor and turnover intention [9, 18]. In addition, studies were conducted on the mediating role of psychological capital between nurses' COVID-19 fear and public health education [16], the relationship between psychological capital and turnover intention [14], and psychological capital's mediating role between perceived stress and posttraumatic stress disorder (PTSD) symptoms [17]. Moreover, it was also found that the turnover intention of nurses increased due to the increase in the workload (ICN, n.d.), fatigue [4], and stress [15] of nurses. However, few research studies have been conducted on the impact of emotional labor and positive psychological capital on turnover intention for nurses caring for COVID-19 patients. Nurses' turnover intention has been an important issue since the COVID-19 pandemic not only in South Korea but also internationally [2–4, 14, 15, 18–20], and their indicators should be identified to reduce it. Emotional labor and positive psychological capital are factors that affect nurses' turnover intention as mentioned earlier. Thus, this study aimed to investigate the impact of emotional labor and psychological capital on the turnover intention of nurses caring for patients with COVID-19 in South Korea at a time when the pandemic is not over.

## 2. Methods

### 2.1. Study Design and Participants

The cross-sectional study design was used. Using convenience sampling, nurses working in three shifts and caring for patients with COVID-19 at a general hospital with more than 500 beds in South Korea were recruited. Sample size was calculated using the G^*∗*^Power 3.1 program [21], with a significance level of 0.05, a medium effect size of 0.15, a power of 0.90, and 10 predictive factors. The required sample size for linear multiple regression was 147, but 170 paper-based questionnaires were distributed. A total of 158 nurses answered, with a survey response rate of 86.5%. However, one was incomplete, and two were duplicate. Ultimately, 155 responses were analyzed.

### 2.2. Instruments

Sociodemographic and working characteristics, such as age, sex, marital status, education, working department, clinical experience, length of experience caring for patients with COVID-19, and experience caring for patients with Middle East respiratory syndrome (MERS) or severe acute respiratory syndrome (SARS), were included in the structured questionnaires.

Emotional labor was assessed using the Emotional Labor Scale for nurses developed by Hong [22]. This 16-item scale consists of three subscales: emotional modulation efforts in the profession (7 items), patient-focused emotional suppression (5 items), and emotional pretense by norms (4 items). The items are scored on a 5-point Likert scale ranging from 1 (“not at all”) to 5 (“very much”), and the total score is within 16–80. High scores indicate greater emotional labor. Cronbach's *α* during the scale development was 0.81 [22], whereas that in this study was 0.87.

Furthermore, positive psychological capital was evaluated using the Korean version of the Psychological Capital Questionnaire 24 (PCQ-24) developed by Luthans et al. [11] and revised by Lim [23] to suit the domestic situation in Korea. This 18-item scale has four subscales: self-efficacy (5 items), optimism (5 items), hope (5 items), and resilience (3 items). The items are scored on a 5-point Likert scale ranging from 1 (“not at all”) to 5 (“very much”); the total score ranges 18–90, with higher scores indicating higher positive psychological capital. Cronbach's *α* during the revised scale was 0.92 [23], whereas that in this study was 0.91.

For measuring nurses' turnover intention, the Korean Nurse Turnover Intention Scale (K-NTIS) developed by Yeun and Kim [24] was used. This 10-item scale consists of three subscales: job satisfaction (4 items), interpersonal relationships (3 items), and work performance (3 items). Each item is scored on a 5-point Likert scale ranging from 1 (“not at all”) to 5 (“very much”); the total score ranges 10–50, with higher scores indicating higher turnover intention. Cronbach's *α* during the scale development was 0.83 [24], whereas that in this study was 0.87.

### 2.3. Data Collection and Ethical Considerations

This study was conducted from March 18 to 27 of 2022 after being approved by the Clinical Trial Review Committee of Kyungpook University Hospital (IRB No.: KNUH 2022-02-014-002). Permission to collect data was obtained upon visiting the nursing departments that managed the quarantine area for patients with COVID-19 (2 intensive care units, 2 wards, and 1 emergency room). Given that the participants worked in three shifts, we visited the corresponding nursing department several times before and after shifts to explain the study's purpose and methods. The researcher explained enough so that each participant could complete the survey only once, and the permission form was prepared individually according to the IRB guidelines. The questionnaire was returned after that to provide sufficient time for the subjects.

Before data collection, written informed consent was obtained from nurses who wanted to participate in this study. The consent form acknowledged that the participants joined the research voluntarily and that they were explained that they could withdraw at any time without disadvantages. Furthermore, all data would be used for academic research purposes, and personal information would remain confidential. The participants received a small gift for completing the survey.

### 2.4. Data Analyses

The data were analyzed using SPSS Statistics 28.0. The participants' emotional labor, positive psychological capital, and turnover intention were measured using descriptive statistics to calculate the means and standard deviations. The differences of turnover intention according to the participants' characteristics were analyzed using a *t*-test and ANOVA. The correlation between variables was evaluated using Pearson's correlation coefficient, and the effects of the participants' emotional labor and positive psychological capital on turnover intention were determined by multiple regression analysis.

## 3. Results

### 3.1. Sociodemographic and Working Characteristics

Table 1 shows the participants' characteristics. Of the 155 participants, 92.9% were females and 72.3% were 22–29 years old, with a mean age of 29.15 years. Regarding marital status, 81.3% were single. In addition, 88.4% held a bachelor's degree or higher. The current place of work was mostly intensive care unit (ICU) (47.7%), followed by wards (33.5%) and emergency room (ER) (18.7%). The mean length of clinical experience was 5.92 years, with 60% participants having less than 5 years. The mean length of experience in caring for patients with COVID-19 was 10.81 months, with 51% under 6 months. Moreover, majority of the participants (94.8%) had no experience caring for patients with an emerging infectious disease such as MERS or SARS.

### 3.2. Descriptive Statistics and Differences on Turnover Intention

The mean scores of emotional labor, positive psychological capital, and turnover intention were 54.52 ± 7.55, 58.03 ± 9.06, and 38.92 ± 6.07, respectively. Table 1 shows the differences in turnover intention according to the participants' characteristics. A significant difference by sex was observed; female nurses had a higher turnover intention score than male nurses (*t* = −3.31; *p* < 0.001). However, no significant differences in turnover intention were noted according to age, marital status, education, department, clinical experience, length of experience caring for patients with COVID-19, and experience of caring for patients with MERS or SARS.

### 3.3. Relationships between Variables

Table 2 shows the results of the correlation analyses of emotional labor, positive psychological capital, and turnover intention. Emotional labor had statistically significant positive correlations with turnover intention (*r* = 0.17; *p*=0.041) and positive psychological capital (*r* = 0.17; *p*=0.041). However, no significant correlation was noted between positive psychological capital and turnover intention.

### 3.4. Multivariable Linear Regression Analysis

Table 3 demonstrates the regression analysis results of the effects of emotional labor and positive psychological capital on turnover intention. Before the regression analysis, the multicollinearity between independent variables was examined. The tolerance ranged between 0.94 and 0.97, and all values were above 0.1. In addition, the variance inflation factor was 1.03–1.07. Thus, no problem in multicollinearity was observed between the independent variables. Moreover, the Durbin–Watson value for the independence test of the residuals was 1.86, which is close to the reference value of 2, implying that autocorrelation problem of the error did not occur.

In controlling the sex variable, which showed a significant difference in turnover intention, a dummy variable was changed. At step 1, sex was a significant impact factor on turnover intention (*β* = 0.26; *p*=0.001). At step 2, sex (*β* = 0.24; *p*=0.003) and emotional labor (*β* = 0.18; *p*=0.019) were affecting turnover intention. In this context, female nurses and having greater emotional labor were associated with a higher turnover intention, and the explanatory power of these variables for turnover intention was 9% (Table 3).

## 4. Discussion

In this study, the mean score of emotional labor among the nurses caring for patients with COVID-19 was 54.52, which is consistent with the previous study that the mean was 55.62 for 171 nurses working at comprehensive nursing care service wards in South Korea [25]. Before or during the COVID-19 pandemic, nurses' emotional labor was above average. During the pandemic, it became severe because of barriers caused by personal protective equipment (PPE) use, low trust relationships, and negative responses from infected patients [6]. For the positive psychological capital of our participants, the score was 58.03. This result was similar to Mubarak et al. [16] study, which the positive psychological capital score was 56.52 for 243 Pakistan nurses during the COVID-19 pandemic. The positive psychological capital is important for managing psychological pain [26] and reducing nurses' fear of COVID-19 [16]; thus, developing measures to increase it is necessary. Regarding the turnover intention of nurses caring for patients with COVID-19, the score was 38.92, which is consistent with the previous study that the score was 31.50 for 174 Korean nurses in 2022 [19]. The higher turnover intention present study may have resulted from the sudden and steep increase in nurses' work, which included wearing PPE while caring for patients with COVID-19, and the large number of patients being hospitalized [4].

Our study results indicated that emotional labor had a significantly positive correlation with positive psychological capital. Nurses with maladaptive cognitive emotional regulation experienced emotional problems, often associated with mental disorders such as depression or anxiety [17]. Nonetheless, possibly, through nurses' emotional labor experience, they were able to quickly recognize their patients' needs and adjust their emotions according to organizational norms, thereby positively influencing their role and job satisfaction improvement and enabling them to efficiently manage situational emotions [27]. The higher the level of psychological capital, the lower their intention in our current study. This is consistent with a previous study involving 4865 nurses from 21 general hospitals randomized in China [14]. Many nurses have complained of mental health-related problems resulting from the prolonged COVID-19 situation [28, 29]; these problems appear to be a factor leading them to leave work. Nurses with a higher level of psychological capital can resolve difficulties and seek external help, and they are more stable at work; thus, their turnover intention is reduced [14].

In this study, sex was the leading factor influencing turnover intention, followed by emotional labor, with 9% explanatory power. At first, female nurses had a higher turnover intention than male nurses. Most previous studies [15, 20, 30] were shown contrary to our result. Mirzaei et al.'s [15] findings reported that male nurses had a higher turnover intention at a hospital in Iran during the COVID-19 outbreak, which might be a different culture of Iran that men having more difficulty tolerating a work setting where it is out of control during an outbreak of an infectious disease. Male nurses reported a greater inclination than female nurses in turnover intention of 1,245 Norwegian nurses [20]. It is a possible explanation on the current result that most nurses are composed of women in the current study and our country, and men should be the head of the family and have financial responsibilities in the Confucian culture. Therefore, sex differences in turnover intention should be further investigated, with equal sex ratios. In addition, intention turnover is different by sex in previous studies [31, 32], and possible explanations were follows: Alsaraireh et al. [31] found that job satisfaction had a negative correlation with turnover intention and that men nurses had a lower job satisfaction and a higher turnover intention than women nurses. In Japan, Minamizono et al. [32] analyzed 328 women nurses and inferred that the higher the burnout score, the higher the intention to change jobs. In other words, considering that the influencing factors of turnover intention vary by sex, increasing job satisfaction in men nurses and lowering burnout including emotional exhaustion in women nurses may reduce turnover intention.

This study showed that emotional labor was a significant factor influencing turnover intention, consistent with Bartram et al.'s [33] findings involving 183 nurses in Australia and 1160 nurses in China [18]. Emotional labor of eleven nurses in the front line against the COVID-19 pandemic in a Portuguese study was analyzed as five themes: challenges experienced by nurses in the front line, emotions experienced by nurses in service care, emotional responses of nurses and patients, emotional labor of nurses in the patient care process, and opportunities for development in the face of the emotional challenge required of nurses in combating COVID-19 [6]. Emotional labor can be overcome through an interpersonal or intrapersonal approach. Emotional support within the team facilitates the emotional labor's performance, which focuses on the patient and the relationship of care with emotional expression or touch, and self-focused emotional labor characterized by positive and adaptive emotional management [6]. In a previous study to verify the effect on nurses' emotional labor, resilience, and self-efficacy by applying an emotional coaching program for nurses, emotional coaching programs were useful for enhancing emotional labor management with 60 nurses who worked at a general hospital [34]. Therefore, emotional labor programs and education to strengthen teamwork may be developed and applied to reduce nurses' intention to resign.

In this study, the relationship between positive psychological capital and turnover intention was not statistically significant, and positive psychological capital was not a factor that can influence turnover intention. These results are not consistent with a previous study in which psychological capital showed a significantly negative correlation with turnover intention for nurses [14]. One probable explanation is that people with high positive psychological capital would find better a working environment and job options any time when they are stressed out at work [35]; thus, it may not be an influence factor. Although our results differed from those of the previous study, psychological capital is essential for minimizing turnover intention [14]. Psychological capital intervention revealed significant improvements and remained stable over 1 month in the psychological capital [36]. In addition, nurse managers conducted psychological capital training courses and established a psychological capital enhancement system to reduce nurses' tendency to leave by improving their psychological capital level [14]. Therefore, psychological capital intervention may be applied to reduce nurses' turnover intention in organizational settings.

### 4.1. Study Limitations

This study has the following limitations: First, the study participants were from a single university hospital in South Korea, with more than 500 beds. Future studies should expand the research area for generalizability. Second, owing to the relatively high proportion of female nurses in the sample, the sex variable was controlled in the regression model to rule out the confounding effects; in future studies, sex should be controlled, or the sex ratio should be equal.

Moreover, given that nurses' turnover intention can affect not only the organization but also personal experiences or relationships with patients, developing a turnover intention-measuring scale that includes organization-related questions as well as questions about nurses' personal experiences and relationships with patients is necessary. However, despite these limitations, this study provides important findings concerning the factors that influence nurses' turnover intention, specifically the effects of sex and emotional labor.

## 5. Conclusions

This study reports the results of a descriptive survey to investigate the levels of emotional labor, positive psychological capital, and turnover intention in nurses caring for patients with COVID-19, clarifying the effects of emotional labor and positive psychological capital on turnover intention. Sex and emotional labor were identified as factors influencing turnover intention, with 9% explanatory power. Therefore, to lower the turnover intention of nurses in charge of patients with COVID-19, hospitals need to develop measures that can reduce their emotional labor.

### 5.1. Implications for Nursing Management

Increasing turnover intention in nurses implies decreasing nursing personnel; consequently, patients may not receive the proper nursing care. As a result of this study, nurses' emotional labor should be reduced to lower the turnover intention; this step can ultimately serve as a countermeasure to the shrinking workforce. Meacham et al. [9] said that high-involvement work practices (HIWPs) and individual resilience can buffer the negative effects of emotional labor on turnover intention of nurses. Based on this, it will be possible to increase HIWPs and develop resilience at the personal level to reduce emotional labor and consequently lower turnover intention. Also, the study findings are useful as foundational or applicable knowledge for developing programs designed to reduce the emotional labor of nurses caring for patients with COVID-19 not only in Korea but also internationally. To retain nurses, nurses and hospital managers should together implement programs aimed at reducing nurses' emotional labor and enhancing psychological capital (e.g., emotional coaching program and psychological capital intervention) and increase the number of nurses.

## Figures and Tables

**Table 1:** Participants' characteristics and differences in turnover intention (*N* = 155).

Variables	Category	*n* (%)	M ± SD	*t*/*F* (*p*)
Sex	Male	11 (7.1)	3.33 ± 0.75	−3.31 (<0.001)
	Female	144 (92.9)	3.94 ± 0.57	

Age (years)	22–29	112 (72.3)	3.92 ± 0.57	0.41 (0.663)
	30–39	31 (20.0)	3.81 ± 0.79	
	40–52	12 (7.7)	3.83 ± 0.61	

Marital status	Single	126 (81.3)	3.90 ± 0.05	0.50 (0.618)
	Married	29 (18.7)	3.84 ± 0.13	

Education	Associate degree	18 (11.6)	3.86 ± 0.17	−0.23 (0.818)
	≥Bachelor	137 (88.4)	3.90 ± 0.05	

Current place of work	Ward	52 (33.5)	3.80 ± 0.08	1.43 (0.244)
	ICU	74 (47.7)	3.90 ± 0.07	
	ER	29 (18.7)	4.04 ± 0.11	

Clinical experience (years)	<5	93 (60.0)	3.90 ± 0.59	2.37 (0.097)
	5–<10	34 (21.9)	4.02 ± 0.46	
	≥10	28 (18.1)	3.89 ± 0.61	

Length of experience caring for patients with COVID-19 (months)	<6	79 (51.0)	3.88 ± 0.60	1.04 (0.357)
	6–<12	16 (10.3)	4.09 ± 0.58	
	≥12	60 (38.7)	3.85 ± 0.62	

Experience caring for patients with MERS or SARS	Yes	8 (5.2)	4.30 ± 0.16	1.97 (0.051)
	No	147 (94.8)	3.87 ± 0.05	

*Note*. COVID-19 = coronavirus disease 2019; MERS = Middle East respiratory syndrome; SARS = severe acute respiratory syndrome.

**Table 2:** Correlations of emotional labor, positive psychological capital, and turnover intention (*N* = 155).

Variables	Emotional labor	Positive psychological capital	Turnover intention
	*r* (*p*)		
Emotional labor	1		
Positive psychological capital	0.17 (0.041)	1	
Turnover intention	0.17 (0.041)	−0.13 (0.120)	1

**Table 3:** Factors affecting turnover intention.

Variables	*B*	SE	*β*	*t*	*p*	*R* ^2^	Adj.*R*^2^	*F* (*p*)
Step 1	3.33	0.18		18.77	<0.001	0.07	0.06	10.94 (0.001)
Sex (male)†	0.61	0.18	0.26	3.31	0.001			
Step 2	2.99	0.48		6.27	<0.001	0.11	0.09	5.94 (0.001)
Sex (male)†	0.56	0.19	0.24	3.04	0.003			
Emotional labor	0.24	0.10	0.18	2.36	0.019			
Positive psychological capital	−0.13	0.10	−0.11	−1.37	0.174			
	*R* ^2^ = 0.11, Adj.*R*^2^ = 0.09, *F* (*p*) = 5.94 (<0.001)							

†Dummy coded.

## Data Availability

The data supporting the results of this study are available on request from the corresponding author. The data are not publicly available due to privacy or ethical restrictions.
